# Patient presenting with lipoma of the index finger: a case report

**DOI:** 10.1186/1757-1626-3-20

**Published:** 2010-01-12

**Authors:** Efstathios Chronopoulos, Nikolaos Ptohis, Christos Karanikas, Alkis Kalliakmanis, Spyros Plessas, Ioannis Neofytou, Fotios Laspas, Ioanna Tzovara, Athanasios Chalazonitis

**Affiliations:** 1 Department of Orthopaedic, Surgery, Athens University, School of Medicine, Konstantopouleio Hospital, Ag Olgas 3-5, N. Ionia 14233, Athens, Greece; 2 Department of Radiology, Alexandra Hospital, Vasilisis Sofias 80, 11528, Athens, Greece

## Abstract

**Introduction:**

Lipomas can be found anywhere in the body with the majority located in the head and neck region as well as in the shoulder and back. They are not very common in the hand and those involving the fingers are very rare. Although, it is not the only case reported, lipoma of the index finger is very uncommon.

**Case presentation:**

A 52-year-old Caucasian man presented with a lipoma of the right index finger. He complained of no pain but he had difficulty in manual movements. Treatment was surgical excision of the lipoma. There has been no recurrence for two years.

**Conclusion:**

Although lipomas of the fingers are rare entities, their awareness is imperative since the differential diagnosis from other soft tissue tumors and from the special lipomatous subtype involved is quite extensive.

## Introduction

Lipomas are benign, mesenchymal neoplasms occurring in areas of abundant adipose tissue [1]. They can be found anywhere in the body with approximately 15-20% located in the head and neck region and the majority of the rest in the shoulder and back [2]. They are not very common in the hand and those involving the fingers are very rare, with reported incidence of 1% [3]. The first patient reported with a lipoma of the finger was by Stein in 1959 [4] and since then, 14 cases were identified in the literature that concerned lipoma case reports of the fingers [5]. Of those, totally 3 were on the index finger, 2 cases occurring distal to the right proximal interphalangeal joint [6] and 1 case to the left index finger [7], all of them postraumatic in nature. We present another case, with no prior traumatic history, a tendon sheath lipoma involving the proximal and middle phalanx of the right index finger.

## Case presentation

**Figure 1:**
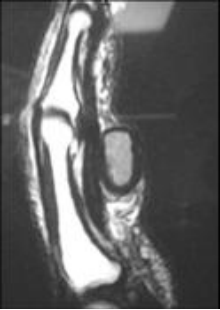
**MRIT1 sequence, saggital image of the lipoma and its attachment to the tendon sheath**.

A 52-year-old Caucasian man was presented with a massive soft tumour of the volar aspect of the proximal and middle phalanx of the right index digit. The mass appeared five years prior to the medical consultation, and was asymptomatic until 12 months ago, when the patient first complained of limitation in the range of motion, especially at flexion and pain during manual maneuvers. On clinical examination he had a soft mobile, elastic mass at the volar aspect of the 2^nd^digit with no disturbance of sensibility. He had no ulceration or pigmentation on the overlying skin, neither any other inflammatory changes such as redness, heat or pain on palpation. Plain anteroposterior radiograph of the digit showed no invasion of the bone, thus excluding parosteal lipoma from the differential diagnosis. Unenhanced MRI shows the exact position and morphology of the lesion in sagittal view, with the characteristic capsule of the mass suggesting a working diagnosis of lipoma (Figure 1).

**Figure 2:**
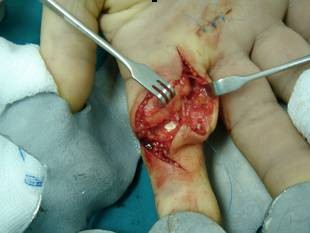
**Lipoma arises from the subcutaneous tissues of proximal phalanx of the right first digit**.

**Figure 3:**
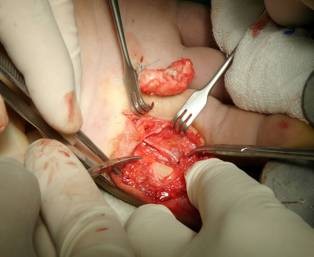
**Removal of the tumor including the extensions into the tendon sheath**.

Under general anesthesia and the use of tourniquet, we performed a 'Z' shape incision in the volar aspect of the finger. The fatty, lobulated mass was found to be localized in the subcutaneous tissue and was attached close to the proximal and middle phalanx of the digit without interfering with the fascicles of the tendon (Figure 2). The neoplasm was removed completely including the extensions into the tendon sheath avoiding damage to the neurovascular bundles (Figure 3). The specimen measured 2 × 1 × 1 cm. Histological examination showed a yellowish, lobulated, ulcerative lipoma confirming the initial diagnosis. No complications occurred during the postoperative period while the patient achieved full range of motion. There were no sings of recurrence after a follow-up of 24 months.

## Discussion

Lipomas are tumors of non-neural origin such as lipofibromatous hamartoma and haemangioma and account for approximately 16% of soft tissue mesenchymal tumors [5]. According to 2002 World Health Organization's committee for the Classification of Soft Tissue Tumors [8], they are categorized into 9 entities, including lipoma, lipomatosis, lipomatosis of nerve, lipoblastoma, angiolipoma, myolipoma of soft tissue, chondroid lipoma, spindle cell/pleomorphic lipoma and hibernoma. Benign lipomatous lesions affecting bone include intraosseous lipoma, parosteal lipoma and liposclerosing myxofibrous tumor. Benign lipomatous lesions may also affect joints and tendon sheaths, either focally as in our case report, or more commonly diffusely (lipoma arborescens).

They are seldom encountered in the hand and are extremely rare in the digits. Lipomas appear mostly in the fifth and sixth decade and probably are the most common solid cellular hand tumors. These lightly encapsulated tumors are composed of mature fatty tissue where the central lipid droplet and peripherally located nucleus forms the characteristic signet ring cell [1]. They arise from mesenchymal primordial fatty tissue cells. These tumors may be superficial, arising from the subcutaneous tissues and or less commonly may be subfascial, arising deep in the palm within the Guyon canal, the carpal tunnel or the deep palmar space [9,10] and generally being of bigger size [11]. Finally, in few cases, they may arise from juxta-articular regions or adjacent to the periosteum (parosteal lipoma), they may erode into the bone and cause focal cortical hyperostoses, osseous projection, subperiosteal new bone formation and bowing of the bone [12,13].

Clinically, superficial lipomas commonly appear as asymptomatic, slow growing, soft fluctuant, and bulging, lobulated and mobile mass. Unless they lie in canals commonly associated with nerve compression, they cause pain and distal sensory changes and motor weakness [13]. Because of their enlarging size, they may lead to limitation of mobility and impairment of grasping. Lateral deviation of the fingers also may be present when the tumor arises around the metacarpophalangeal joints. Clinical evaluation of superficial lipomas is accurate for diagnosis in up to 85% of cases [14], contrary to deep lipomas for which clinical evaluation indicates only a nonspecific mass.

Radiological evaluation is diagnostic in up to 71% of cases [14]. Computed tomography and especially magnetic resonance imaging are helpful in the assessment of such lesions. The MR images reveal of such lesions reveals tissue that is isointense relative to subcutaneous fat, regardless of pulse sequences. When contrast is applied, the mass does not enhance except for its capsule. In 37-49% of cases CT or MR images reveal intrinsic thin septa (< 2 mm), a sign that is considered almost pathognomonic for the diagnosis of lipoma [15]. The main imaging criteria used to differentiate those lesions from their malignant counterparts, liposarcomas, are the absence of septa in most of the cases, the presence of mineralization areas best depicted with CT and the absence of interdigitations with skeletal muscle, a feature described only in intramuscular lipomas [14].

Lipomas arising from the deep palmar space tend to present in the periphery of the hand because of the unyielding nature of the overlying palmar aponeurosis. Diagnosis in the hand and digits can be difficult, because of their rarity and deeper location. The differential diagnosis as mentioned, includes other soft tissue tumors such as ganglion cysts, giant-cell tumors, myxomas, angiolipomas and intraneural lipofibromas [7]. However, as in our case, their proximity and extension to the tendon sheath along with the imaging features, gives a clue to the proper characterization as a lipoma and the identification of the specific subtype. Lipomas of the tendon sheath, being very rare manifest with two variants: (a) a discrete solid mass of fatty tissue and (b) a lipoma-like lesion composed of hypertrophic synovial villi distended with fat and called lipoma arborescens [14]. The latter are more common and occur ussually in the knee while the former, an example of which is probably our case, is less common and tends to occur in hand and wrist.

Careful dissection is necessary during the surgical procedure in order to avoid recurrence. Recurrence after marginal excision is less than 5%. In our case the tumor located subcutaneously, with a part extended into the tendon sheath. Clinical diagnosis was confirmed with histological examination. Histological examination showed a yellowish, lobulated, ulcerative lipoma confirming the initial diagnosis but unfortunately the picture is not available, although it was thoroughly searched in all involved departments. No complications occurred during the postoperative period while the patient achieved full range of motion. There were no sings of recurrence after a follow-up of 24 months. Surgical dissection and removal of the neoplasm was performed with no post-operative complications and is the treatment of choice.

## Conclusion

Although lipomas of the fingers are rare entities, their awareness is imperative since the differential diagnosis from other soft tissue tumors and from the special lipomatous subtype involved is quite extensive.

## Consent

All reasonable attempts to gain consent have been made. The patient is anonymous and there is no reason to think that the patient or their family would object to publication.

## Competing interests

The authors declare that they have no competing interests.

## Authors' contributions

CE, KA and PA are the orthopedic surgeons that contributed to the excision of the lipoma in the OR. NP and KC were the authors that contributed mostly to the medical writing and formatting of the article and finally NI, LF, TI and CA were the members of the radiology department that made the diagnosis and the thorough literature research.
